# Retrospective surgical outcomes of Gartland type III supracondylar humeral fractures with absent pulse in children

**DOI:** 10.3389/fped.2026.1836881

**Published:** 2026-06-15

**Authors:** Maria Rizzo, Sabrina Carbone, Michela Saracco, Fabio Spinetti, Emanuela Asunis, Gianluca Colella, Anna Petrone, Gaetano Mignano, Fabio Schiano, Liberato Carbone, Massimo Mariconda

**Affiliations:** 1 Department of Public Health, Orthopedics and Traumatology Unit, University “Federico II” of Naples, Naples, Italy; 2 Department of Orthopaedic Surgery, AORN Santobono Pausilipon Children’s Hospital, Naples, Italy; 3 Department of Vascular Surgery, Ospedale del Mare, Naples, Italy

**Keywords:** brachial artery, Gartland type III, neurovascular injury, pediatric trauma, pink pulseless hand, pulseless limb, supracondylar humeral fractures in children, vascular injury

## Abstract

**Introduction:**

Supracondylar humeral fractures are the most common elbow injuries in children. Gartland type III fractures require urgent treatment due to the high risk of neurovascular injury. Vascular compromise occurs in 5%–15% of cases, most commonly involving the brachial artery, and clinical presentation ranges from complete ischemia (“cold, pale hand”) to the “pink pulseless hand” (PPH).

**Materials and methods:**

Twelve children (9 males, 3 females) with Gartland type III supracondylar humeral fractures and absence of a palpable radial pulse, treated between April 2023 and September 2025, were retrospectively analyzed. All patients underwent open reduction and pin fixation within 12 h of injury. Vascular management was guided by limb perfusion: ischemic limbs underwent urgent vascular exploration, whereas patients with PPH were managed with close clinical and instrumental monitoring. Outcomes were assessed using Flynn criteria.

**Results:**

The mean age was 5.75 ± 2.38 years, with a mean follow-up of 6 ± 4 months. Ten patients presented with ischemia and underwent vascular exploration, while two patients with PPH were managed without vascular intervention. Although overall outcomes were satisfactory, angular deformities occurred in 50% of patients (three varus, three valgus), and only 50% achieved an excellent Flynn result. Complications included anterior interosseous nerve neuropraxia (two cases), brachial artery thrombosis (one case), and compartment syndrome (one case). Comparisons between groups were exploratory and did not reach statistical significance.

**Conclusion:**

Management should be guided by limb perfusion rather than pulse status alone. Patients with preserved perfusion may be safely observed, whereas ischemic limbs require urgent intervention. These injuries should be considered within a neurovascular spectrum. Given the small sample size and short follow-up, findings are descriptive and require confirmation in larger studies.

## Introduction

Supracondylar fractures of the humerus are among the most common elbow injuries in the pediatric population, accounting for approximately 55%–75% of all fractures at this level (1). Among these, Gartland type III fractures, defined as completely displaced, require urgent treatment due to the increased risk of neurovascular injury (2). Vascular compromise occurs in approximately 5%–15% of cases and most commonly involves the brachial artery, which may be compressed, stretched, occluded, or thrombosed as a result of fracture displacement (3, 4).

Clinical presentation ranges from complete limb ischemia (the “cold, pale hand”) to the so-called “pink pulseless hand” (PPH), in which collateral circulation is sufficient to maintain apparent peripheral perfusion despite the absence of a palpable radial pulse (5). When vascular compromise is suspected, diagnostic evaluation may include computed tomography angiography and duplex Doppler ultrasound, which are useful for assessing arterial flow and guiding management decisions (6).

However, optimal management remains controversial and is largely dependent on clinical presentation. In particular, the management of PPH following elbow trauma is still debated. Some authors advocate routine brachial artery exploration (6), whereas others suggest that careful observation is safe in the presence of detectable Doppler signals at the wrist (7). Nonoperative management is generally based on the assumption that PPH results from transient arterial spasm; however, the exact mechanism of arterial compromise remains unclear.

In contrast, the presence of a cold PPH—often associated with reduced peripheral oxygen saturation and absent Doppler signals—requires urgent vascular surgical intervention, sometimes combined with decompressive fasciotomy to prevent compartment syndrome (8, 9).

The aim of this study is to describe our clinical experience in the management of this case series.

## Materials and methods

This retrospective single-center study included twelve children (9 males and 3 females) who presented to the Emergency Department of Santobono Pausilipon Children's Hospital (Naples, Italy) between April 2023 and September 2025 with closed Gartland type III supracondylar humeral fractures and absence of a palpable radial pulse.

Patients with fractures other than Gartland type III, open fractures, preoperative nerve injuries, a history of previous elbow fracture or surgery, or multiple injuries due to polytrauma were excluded.

At admission, each patient underwent a standardized clinical evaluation to assess signs of upper limb vascular compromise, including absence of the radial pulse and reduced peripheral perfusion, manifested by a cold or pale hand. A management algorithm based on our institutional protocol was applied (Figure 3). In cases of uncertain or particularly severe clinical presentation, additional imaging studies, including computed tomography angiography or color Doppler ultrasonography, were performed to better define vascular status.

All patients underwent surgical treatment consisting of open reduction and pin fixation within 12 h of injury.

Limb perfusion was assessed using clinical parameters, including skin color, temperature, capillary refill time, and peripheral oxygen saturation measured by pulse oximetry.

Vascular management was guided by clinical presentation. Patients presenting with signs of acute ischemia underwent immediate surgical exploration of the brachial artery in addition to fracture fixation. In contrast, patients with a pink pulseless hand were managed conservatively with close clinical and instrumental monitoring, including pulse oximetry and serial Doppler ultrasound examinations, without vascular exploration.

Postoperatively, a brachiometacarpal plaster immobilization was applied for approximately 30 days and removed after radiographic confirmation of callus formation on standard anteroposterior and lateral views. Kirschner wires were removed as a day-case procedure without sedation.

Outcomes were assessed according to Flynn criteria (10), with cosmetic results evaluated based on loss of carrying angle and functional outcomes based on loss of range of motion. Patient demographic characteristics, vascular presentation, angular deformities, and complications are summarized in Table 2.

Written informed consent was obtained from the patients' legal guardians for the publication of any potentially identifiable images or data included in this article.

### Statistical analysis

Statistical analysis was performed using SPSS software (IBM Statistics), and data were collected and organized using Microsoft Excel. Categorical variables are presented as frequencies and percentages, while ordinal variables are reported as medians and ranges.

Given the small sample size and the unbalanced distribution between vascular presentation groups, nonparametric and exact statistical tests were applied.

The association between vascular presentation (ischemia vs. pink pulseless hand) and ordinal outcomes, including Flynn functional score and angular deviation according to Flynn criteria, was assessed using the Mann–Whitney *U*-test. Associations between vascular presentation and categorical variables, including the presence of angular deformity (varus, valgus, or none) and neurovascular complications, were evaluated using Fisher's exact test.

A *p*-value < 0.05 was considered statistically significant.

## Results

We retrospectively enrolled twelve patients with Gartland type III supracondylar humeral fractures who underwent surgical treatment between 2023 and 2025. The mean age at the time of surgery was 5.75 ± 2.38 years (range 3–10 years).

All patients presented to the Emergency Department following low-energy trauma, most commonly accidental falls at home or during play. At initial clinical assessment, all patients had absence of a palpable radial pulse, which constituted the primary indication for urgent management.

**Figure 1:**
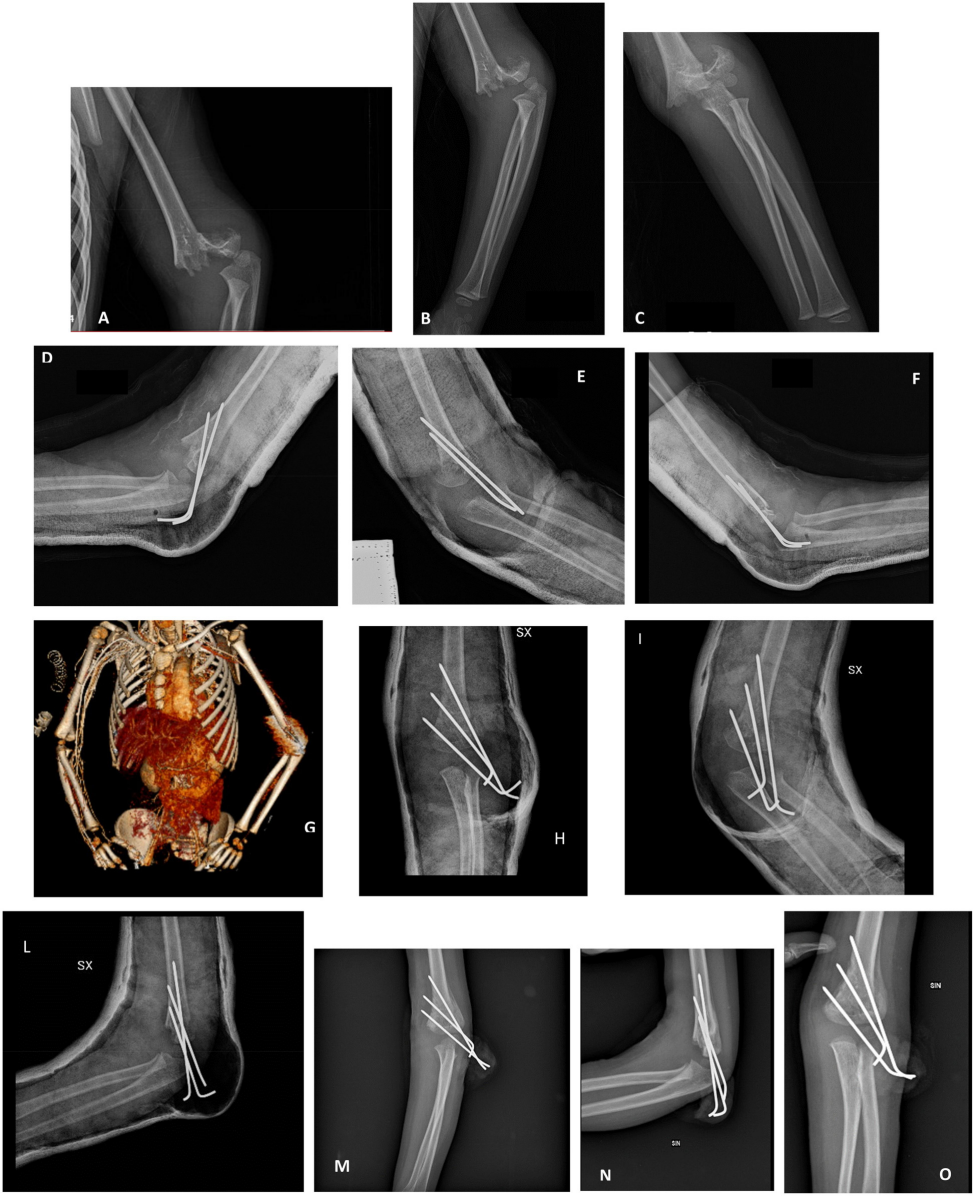
**(A–C)** 5-year-old male. Initial x-rays showed a Gartland type III supracondylar humeral fracture. On physical examination, deformity of the anatomical profile and functional impairment were observed. The radial pulse was present at admission, and a positive Kirmission sign was noted. The patient was poorly cooperative during the initial clinical evaluation. Given the high-energy mechanism of injury, an abdominal ultrasound and surgical evaluation were performed in the Emergency Department prior to admission to the Orthopedic Department. Approximately 12 h after admission, the radial pulse was no longer palpable on digital examination. Clinical assessment revealed anterior interosseous nerve deficit, characterized by inability to flex the interphalangeal joint of the thumb and the distal interphalangeal joint of the index finger, associated with hypoesthesia of the index finger. (**D—F)** Postoperative radiographs demonstrated two lateral, divergently placed Kirschner wires with bicortical fixation, providing stable fixation of both columns without convergence or crossing at the fracture site. The limb was then immobilized with a dorsal brachiometacarpal splint. **(G)** Contrast-enhanced CT angiography showed demonstrated occlusion of the distal brachial artery. **(H—L)** A surgical revision procedure was performed one week after initial presentation to optimize fracture fixation. Radiographic evaluation confirmed improved alignment and stabilization with three laterally inserted Kirschner wires. **(M—O)** At 1-month follow-up, callus formation and physiological remodeling were observed. Mild joint stiffness was noted. K-wires and cast were removed, and progressive mobilization was initiated.

**Figure 2:**
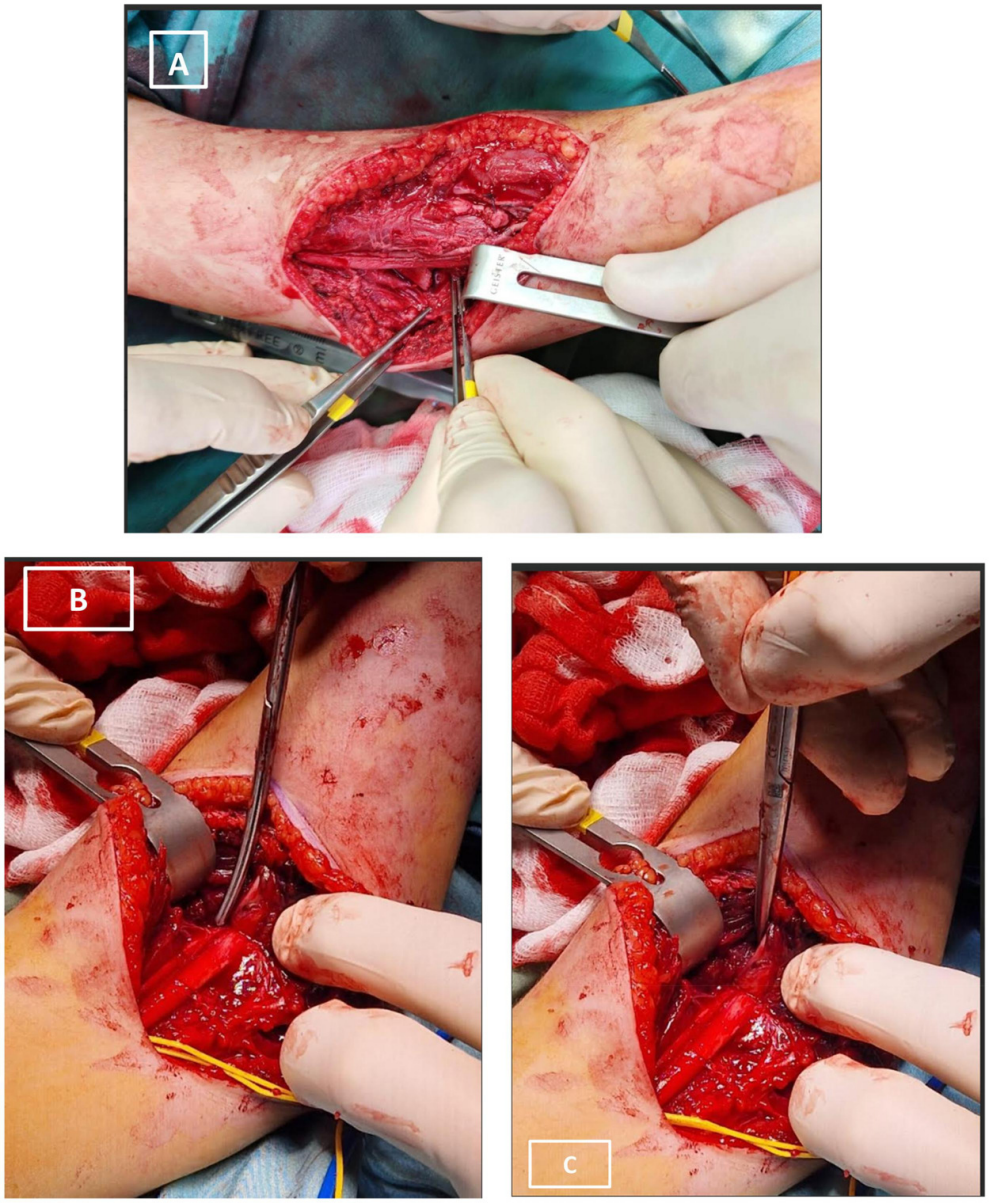
**(A—C)** Vascular presentation of the case illustrated in Figure 1. Open surgical exploration of the brachial artery was performed at the level of the elbow and distal arm.

Ten patients presented with a cold, pale hand and clear signs of acute ischemia. These patients underwent immediate surgical treatment with the involvement of the vascular surgery team. Preoperative imaging (computed tomography angiography or color Doppler ultrasound) confirmed abnormalities of brachial artery flow. Surgical management included evacuation of the perivascular hematoma and release and debridement of the contused brachial artery entrapped within the fracture site. Representative radiographic, angiographic, and postoperative findings are shown in Figures 1 and 2.

The remaining two patients presented with a “pink pulseless hand”. In these cases, fracture fixation was performed without vascular exploration, and a conservative vascular approach with close monitoring was adopted, including pulse oximetry and serial Doppler assessments. Both patients showed prolonged capillary refill time and reduced peripheral oxygen saturation.

**Figure 3:**
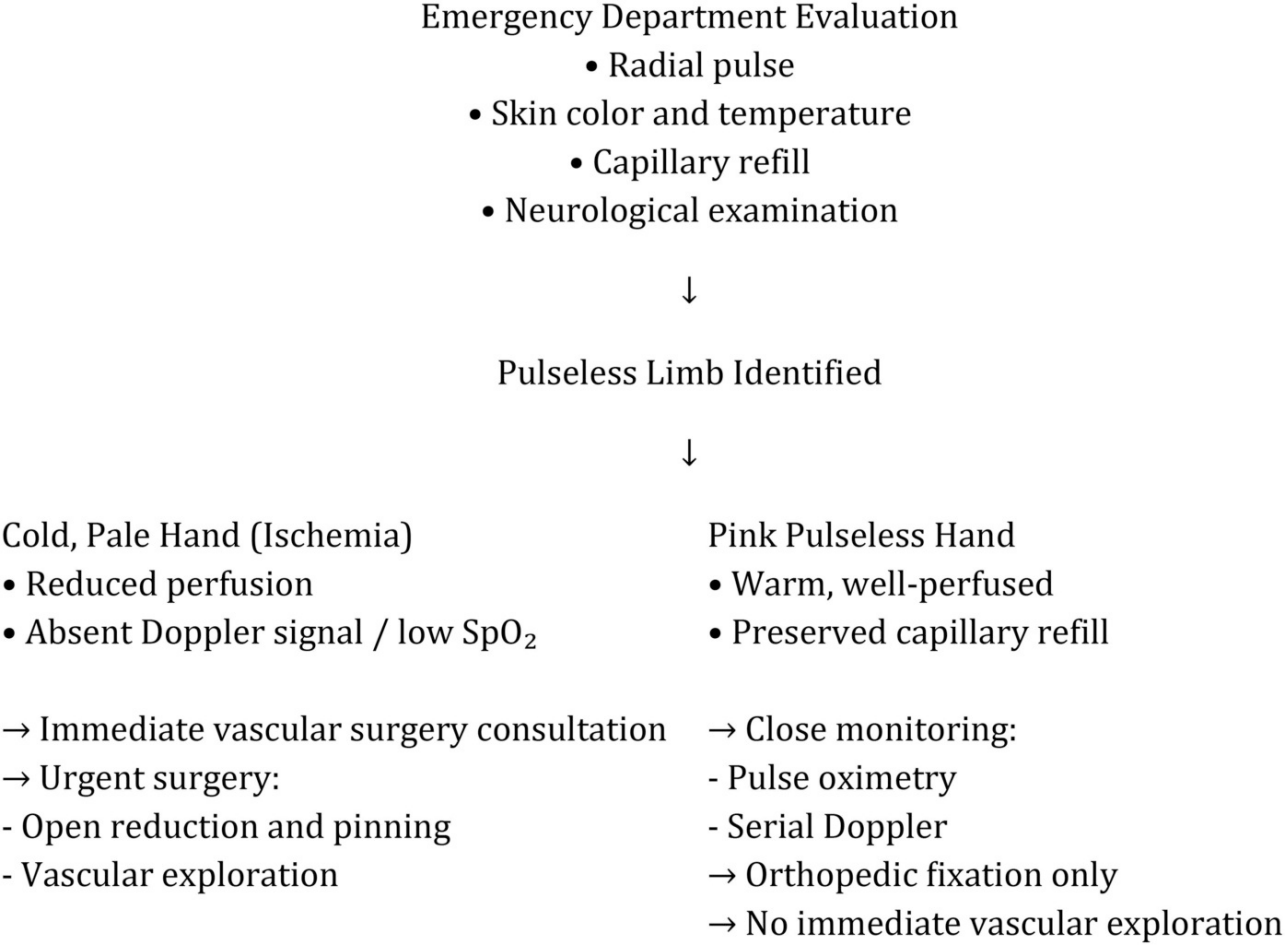
Clinical management algorithm pulseless supracondylar humeral fractures in children.

All patients were managed according to a standardized institutional algorithm (Figure 3). Clinical evaluation included assessment of radial pulse, limb perfusion (skin color, temperature, and capillary refill), and neurological status. When vascular compromise was suspected or findings were equivocal, further imaging with Doppler ultrasound or CT angiography was performed.

Kirschner wires were removed at 4 weeks after radiographic confirmation of fracture healing. No cases of secondary displacement, pin tract infection, wire migration, or iatrogenic ulnar nerve injury were observed. Patients were followed for a mean of 6 ± 4 months. Clinical and functional outcomes were assessed according to Flynn criteria (Table 1).

**Table 1A:** Flynn's functional criteria.

Outcome	*N* = 12
Excellent	6
Fair	3
Poor	3

**Table 1B:** Flynn's clinical criteria (angular deviation).

Outcome	*N* = 12
Excellent	10
Good	2

**Table 2:** Patient level table: vascular presentation was classified as ischemic limb (cold, pale hand) or pink pulseless hand (PPH). Angular deformity was classified as cubitus varus, cubitus valgus, or none.

Patient	Sex	Age	Side	Vascular prsentation	Angular deformity	Complications
1	M	9	Left	Ischemia	Valgus	AIN neuropraxia
2	M	5	Left	Ischemia	None	None
3	M	3	Left	Pink pulseless	None	AIN neuropraxia
4	F	10	Left	Pink pulseless	Varus	None
5	F	6	Right	Ischemia	None	Vascular occlusion
6	M	8	Left	Ischemia	Varus	None
7	M	7	Right	Ischemia	Varus	None
8	M	4	Left	Ischemia	Valgus	None
9	F	3	Right	Ischemia	None	compartment syndrome
10	M	6	Left	Ischemia	None	Brachial artery thrombosis (treated with Fogarty thrombectomy)
11	M	3	Right	Ischemia	Valgus	None
12	M	5	Left	Ischemia	None	None

Patients with a pink pulseless hand showed descriptively worse functional and cosmetic outcomes compared with those presenting with ischemia; however, these differences were not statistically significant (Flynn functional score: *p* = 0.200; angular deviation: *p* = 0.246). These comparisons should be considered exploratory.

No statistically significant association was found between vascular presentation and neurovascular complications (Fisher's exact test, *p* = 1.000), nor between vascular presentation and the type of angular deformity (varus, valgus, or none).

Although overall outcomes were generally satisfactory, angular deformities were observed in 50% of patients, including cubitus varus in three cases and cubitus valgus in three cases. Only 50% of patients achieved an excellent result according to Flynn criteria.

Anterior interosseous nerve neuropraxia occurred in two patients; one presented with impaired flexion of the interphalangeal joint of the thumb. Vascular complications included one case of brachial artery thrombosis, successfully treated with Fogarty thrombectomy, and one case of compartment syndrome. No K-wire–related infections were observed.

## Discussion

Supracondylar humeral fractures in children, particularly Gartland type III injuries associated with vascular compromise, represent a significant clinical challenge. As originally highlighted by Gartland, fracture severity is a key determinant of the risk of neurovascular injury, requiring prompt and appropriate management to prevent irreversible damage.

In our series, not all patients presenting with an absent radial pulse required immediate vascular exploration. In cases of a PPH with preserved peripheral perfusion, confirmed by pulse oximetry and Doppler ultrasound, a conservative approach with close clinical and instrumental monitoring was adopted. This strategy is consistent with previous reports suggesting that, in the presence of adequate perfusion, observation may be safe and can avoid unnecessary surgical intervention (16).

Conversely, patients presenting with a cold, pale hand and signs of acute ischemia required urgent surgical management in collaboration with vascular surgeons to restore arterial flow and prevent severe complications such as compartment syndrome or tissue necrosis (17). In our experience, this approach resulted in satisfactory restoration of limb perfusion and generally favorable outcomes. The proposed management algorithm reflects our institutional practice and should be interpreted as a practical approach rather than a universal guideline.

Despite overall functional outcomes were acceptable, our results highlight a non-negligible rate of residual deformities. Angular deformities were observed in 50% of patients, and only half of the cohort achieved an excellent Flynn outcome. These findings underline the severity of Gartland type III fractures with vascular compromise and their potential for suboptimal outcomes. Neurovascular complications were also observed, including anterior interosseous nerve neuropraxia, one case of brachial artery thrombosis and one case of compartment syndrome. These findings highlight the intrinsic severity of these injuries and the importance of early diagnosis and appropriate management.

Although patients with PPH showed descriptively worse functional and cosmetic outcomes compared with those presenting with ischemia, these differences were not statistically significant. This likely reflects the small sample size and limited statistical power. Indeed, the comparison between the two groups was performed using the Mann–Whitney U test, a nonparametric method appropriate for small samples and ordinal variables such as Flynn criteria, which may reduce the ability to detect statistically significant differences. Therefore, the absence of statistical significance should be interpreted with caution and not as evidence of equivalence between groups.

Early surgical intervention is widely considered crucial in cases of vascular compromise. Previous studies, including that by Chaturvedi et al. (11), have reported a negative correlation between treatment delay and functional outcomes, underscoring the importance of timely management.

However, our findings partially differ from those reported by Chaturvedi et al. (11), who identified limb perfusion as the main prognostic factor and demonstrated better functional outcomes in patients with a PPH compared with those with impaired perfusion. In their study, parameters such as capillary refill time and peripheral oxygen saturation were more reliable predictors than the mere presence of a peripheral pulse.

The discrepancy between our results and those of Chaturvedi et al. may be explained by the limited sample size of our cohort, particularly the small number of patients presenting with a PPH, which reduces the statistical power to detect significant differences.

Similarly, no significant association was observed between vascular presentation and the occurrence of neurovascular complications or angular deformities. These findings should be interpreted with caution, as the absence of statistical significance may reflect the small sample size and imbalance between groups rather than true clinical equivalence.

Overall, our results support a management strategy based on careful assessment of limb perfusion rather than the sole presence or absence of a radial pulse. Patients with preserved perfusion may be safely managed with close observation, whereas those with signs of ischemia require urgent surgical intervention. Further studies with larger cohorts are needed to better define prognostic factors and optimize treatment strategies in this complex clinical scenario.

The decision to perform surgery should be made carefully, balancing potential risks and benefits and considering the overall clinical and instrumental findings. Surgical indication should be assessed on a case-by-case basis: intervention is required in cases of acute ischemia and clear vascular compromise, whereas close monitoring may be sufficient in patients with a well-perfused PPH without signs of critical ischemia, as supported by the literature (5, 12). This approach helps to avoid unnecessary invasive procedures and reduces the risk of iatrogenic complications. However, surgical exploration of the vascular bundle is not without potential drawbacks. Vascular exploration may increase operative time and require more extensive soft tissue dissection, potentially leading to a higher risk of infection, scarring, and iatrogenic neurovascular injury. In addition, extensive surgical exposure may negatively affect postoperative elbow mobility and functional range of motion, particularly in pediatric patients.

Therefore, the decision to perform vascular exploration should be carefully balanced against these potential risks, reinforcing the importance of a selective approach based on clinical perfusion rather than routine exploration in all cases.

Supracondylar humeral fractures with vascular compromise should not be regarded solely as vascular injuries but rather as part of a combined neurovascular spectrum. Accordingly, clinical assessment should include both vascular and neurological evaluation, given the close anatomical relationship between these structures (13).

Neurological injuries occur in 6.5%–19% of displaced fractures and most commonly involve the anterior interosseous nerve, particularly in extension-type injuries (14). These injuries are frequently associated with fracture displacement and soft tissue damage and often coexist with vascular compromise.

Recent evidence suggests that arterial injury significantly increases the risk of associated peripheral nerve damage, supporting the concept of a combined neurovascular injury pattern rather than two independent complications. This association likely reflects the severity of the initial trauma, in which displaced fracture fragments may simultaneously compress, entrap, or stretch both vascular and neural structures. In this context, a pulseless limb—especially when associated with neurological deficits—should raise suspicion of a more severe injury requiring careful evaluation and, in selected cases, surgical exploration (15, 18).

In our series, the occurrence of anterior interosseous nerve neuropraxia supports the concept of a combined neurovascular injury pattern. Although most nerve injuries are neurapraxias with favorable recovery, persistent deficits may indicate nerve entrapment or more severe damage (14). Therefore, management should be guided by an integrated neurovascular assessment rather than vascular status alone.

This study has several strengths. First, it focuses on a well-defined and clinically relevant subgroup of pediatric supracondylar humeral fractures (Gartland type III injuries with vascular compromise) which remain a challenging and controversial condition in orthopedic practice. Second, it includes a homogeneous cohort of patients treated at a single pediatric center using a standardized diagnostic and therapeutic protocol, ensuring consistency in clinical assessment, surgical management, and follow-up.

In addition, both functional and cosmetic outcomes were systematically evaluated using Flynn criteria, allowing a comprehensive assessment of clinical results. The study also provides a detailed analysis of vascular presentation and its relationship with outcomes and complications, supported by statistical methods appropriate for small sample sizes.

This study has several limitations that should be acknowledged. The primary limitation is the small sample size, particularly the very limited number of patients presenting with a PPH, which reduces statistical power and the ability to detect meaningful differences between groups. In addition, the retrospective single-center design may introduce selection bias and limit the generalizability of the findings.

Another limitation is the relatively short follow-up period. With a mean follow-up of 6 ± 4 months, and some patients followed for as little as 2 months, the assessment of angular deformities and remodeling potential remains incomplete. In pediatric supracondylar fractures, bone remodeling continues over time, and early alignment outcomes may not reflect final results. Furthermore, although standardized criteria were used, functional outcomes such as the Flynn classification are inherently operator-dependent and may introduce some degree of subjectivity.

Given these limitations, the present study should be interpreted as a descriptive case series, and the findings considered exploratory and hypothesis-generating rather than definitive. Larger prospective studies with longer follow-up are needed to confirm these results and to better evaluate the progression of angular deformities and remodeling potential in this patient population.

## Data Availability

The original contributions presented in the study are included in the article/Supplementary Material, further inquiries can be directed to the corresponding author.
